# Human T Cell Responses to Flavivirus Vaccines

**DOI:** 10.1002/eji.70027

**Published:** 2025-08-17

**Authors:** David Wullimann, Hans‐Gustaf Ljunggren

**Affiliations:** ^1^ Center For Infectious Medicine Department of Medicine Huddinge Karolinska Institutet Stockholm Sweden

**Keywords:** CD4+ T cells, CD8+ T cells, dengue virus, flavivirus, Japanese encephalitis virus, immune memory, tick‐borne encephalitis virus, vaccination, viral protection, yellow fever virus, Zika virus

## Abstract

Flaviviruses are major human pathogens that continue to pose a global health threat, and vaccination is an effective strategy to protect against disease from several flaviviruses. Flavivirus vaccines are believed to confer protection primarily through antibody responses; however, the role of T cells in vaccine immunity remains less explored despite demonstrated contribution in the response to natural infection. This review examines T cell responses induced by licensed or developing flavivirus vaccines, their contribution to protection, and key findings highlighting the importance of cellular immunity. We discuss the role of memory T cells, including CD4+ and CD8+ subsets, in flavivirus vaccine‐induced immunity and compare the immunogenicity of live attenuated versus inactivated vaccines. We also discuss the significance of T cell immunity, cross‐reactivity, and vaccine platform design in shaping durable and broad protection. Additionally, we broaden the discussion toward other human RNA viruses, including the influenza virus and severe acute respiratory syndrome coronavirus 2 (SARS‐CoV‐2). A better understanding of the role of T cell immunity will be essential for optimizing the use of current flavivirus vaccines and developing next‐generation approaches capable of providing long‐lasting immunity against emerging and re‐emerging flavivirus threats.

## Graphical abstract

This review summarises current knowledge of T cell responses induced by flavivirus vaccines being licensed or under development. Existing vaccines against flaviviruses have been developed and analysed primarily in the context of antibody responses, while the role of T cells in contributing to immunity against flaviviruses is less studied.

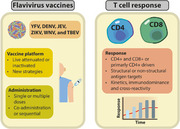

**Abbreviations**
ADE: antibody‐dependent enhancement
APC: antigen‐presenting cell
DENV: dengue virus
ELISpot: enzyme‐linked immunosorbent spot
IFN: interferon
JEV: Japanese encephalitis virus
MHC: major histocompatibility complex
PRR: pattern recognition receptor
SARS‐CoV‐2: severe acute respiratory syndrome coronavirus 2
TBEV: tick‐borne encephalitis virus
TLR: Toll‐like receptor
TNF: tumour necrosis factor
WNV: West Nile virus
YFV: yellow fever virus
ZIKV: Zika virus

## 1 Introduction

Flaviviruses of the *Flaviviridae* family are small, enveloped viruses with positive‐sense RNA genomes of approximately 9 to 13 kb. The *Orthoflavivirus* genus comprises over 50 species found worldwide, most of which are arthropod‐borne [1]. Several of these are major human pathogens, forming distinct groups based on their transmission by either mosquitoes or ticks. These include the mosquito‐borne yellow fever virus (YFV), Zika virus (ZIKV), Japanese encephalitis virus (JEV), dengue virus (DENV), and West Nile virus (WNV), as well as tick‐borne encephalitis virus (TBEV). Collectively, mosquito‐borne flaviviruses cause millions of infections annually in tropical regions of South and Southeast Asia, South America, and Africa, while TBEV is prevalent in Europe and northern Asia. In 2021, an estimated 60 million infections by DENV, YFV, and ZIKV occurred across 124 countries [2]. The continued emergence and spread of flaviviruses pose an ongoing threat to global public health [3].

Vaccination is an effective strategy for preventing flavivirus‐related disease [4]. Licensed vaccines are available for several flaviviruses, including live attenuated vaccines (YFV, DENV, and JEV) and inactivated whole virus vaccines (TBEV and JEV). However, for certain medically significant flaviviruses, such as ZIKV and WNV, no licensed vaccines are currently available despite extensive development efforts. Most vaccines, including those against flaviviruses, have been developed with the primary goal of inducing a humoral response, with neutralizing antibodies serving as the primary correlate of protection [5]. However, protective immunity against flavivirus infections is complex and involves multiple components of the immune system, including T cells. Increasing evidence suggests that vaccine‐induced T cell immunity plays a critical role in modulating the immune response, influencing both the magnitude and durability of protection. In this review, we examine how flavivirus vaccines induce T cell responses. We furthermore discuss their potential role in protection and highlight recent findings that underscore the importance of cellular immunity in vaccine‐mediated protection against flavivirus‐caused diseases. We focus on clinical studies conducted on the most medically significant flaviviruses within the *Orthoflavivirus* genus, including YFV, DENV, JEV, ZIKV, WNV, and TBEV.

### 1.1 Structure of Flaviviruses

**FIGURE 1:**
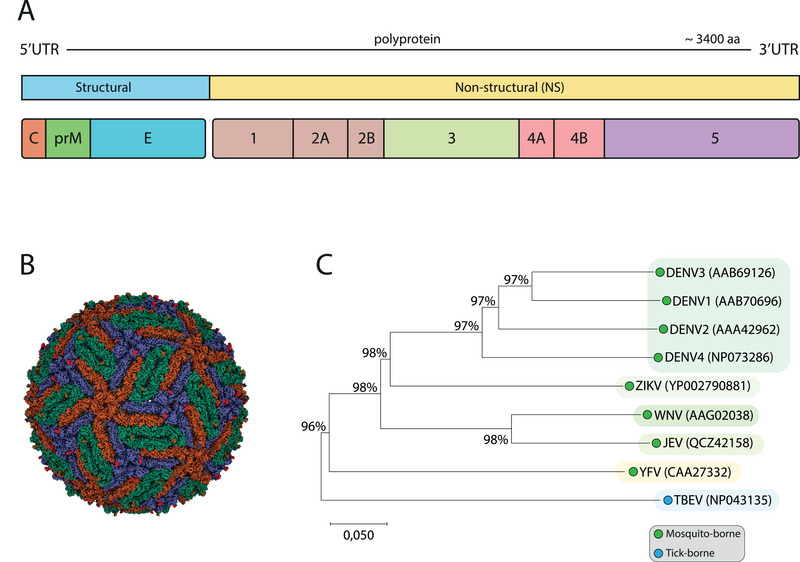
Structure and phylogenetic relationship of flaviviruses. (A) Schematic illustration of the single open reading frame, which is translated into a polyprotein of approximately 3400 amino acids. The major structural and non‐structural proteins are shown (minor accessory proteins are excluded). (B) A 3D rendering of the cryo‐EM structure of the Zika virus at 3.8 Å, retrieved from the PDB using ID 5IRE. (C) Phylogenetic tree of six flaviviruses. The MEGA12 software was used to perform a ClustalW multiple sequence alignment. The phylogenetic tree was constructed using the Neighbor‐Joining method and the p‐distance metric to compute evolutionary distances, where units represent the number of amino acid differences per site. Accession IDs are listed in parentheses.

The flavivirus genome encodes a single open reading frame, which is translated into a polyprotein consisting of three structural proteins and seven non‐structural proteins (Figure 1A). The flavivirus virion is spherical, approximately 50 nm in diameter, and consists of three structural proteins: capsid (C), envelope (E), and either pre‐membrane (prM) in immature virions or membrane (M) in mature virions (Figure 1B). In some cases, infection results in the release of not just mature but also immature virions [6]. The envelope protein is the dominant structural component on the viral surface and plays a key role in receptor binding and viral entry [7]. Each monomer of the envelope protein is composed of three domains (I‐III), along with a stem and transmembrane region. Domains I and II contain multiple highly conserved regions, including the fusion loop [8], whereas Domain III, exposed on the viral surface, contains more variable regions such as receptor‐binding sites [9]. Infected cells synthesize seven non‐structural (NS) proteins with enzymatic activity and are involved in all steps of the viral life cycle: NS1, NS2A, NS2B, NS3, NS4A, NS4B, and NS5. The NS1 protein is unique among flavivirus proteins, as it exists both in a cell‐associated form, which is involved in viral RNA replication, and a secreted form, which regulates complement activation [10]. The NS3 protein contains an RNA helicase domain essential for RNA replication, while NS5 is the largest and a highly conserved flavivirus protein, functioning as the viral RNA‐dependent RNA polymerase (RdRp) [11].

The phylogenetic relationship between flaviviruses is important to consider when evaluating immune responses. All orthoflaviviruses are serologically related, as demonstrated by the high degree of cross‐reactivity observed in antibody binding assays such as ELISA [12]. For diagnostic purposes, neutralization assays are used to determine the specific flavivirus responsible for infection. The envelope protein is the primary target for neutralizing antibodies, and antigenic sites have been mapped across all three structural domains [13]. Although many RNA structures and functions are conserved across orthoflaviviruses, variations in genome length and organization exist, particularly between mosquito‐borne and tick‐borne flaviviruses (Figure 1C).

### 1.2 T Cell Targets Against Flaviviruses

Antigen processing and presentation are the critical first steps in generating effector T cells against viral targets. It involves processing viral antigens into peptides, which bind to MHC molecules and form complexes recognized by the T cell receptor (TCR) [14]. During antigen presentation, CD4+ T cells are primarily activated by peptides with lengths in the range of 12–20 amino acids, which are bound and displayed by MHC class II molecules on professional antigen‐presenting cells. By contrast, CD8+ T cells recognize and are activated by shorter peptides, typically 9–12 amino acids in length, presented by MHC class I molecules on all nucleated cells. Accurately identifying and characterizing the epitopes targeted by virus‐specific T cells is crucial, as it forms a foundational step in immunophenotyping these immune responses and elucidating T cell functionality.

**FIGURE 2:**
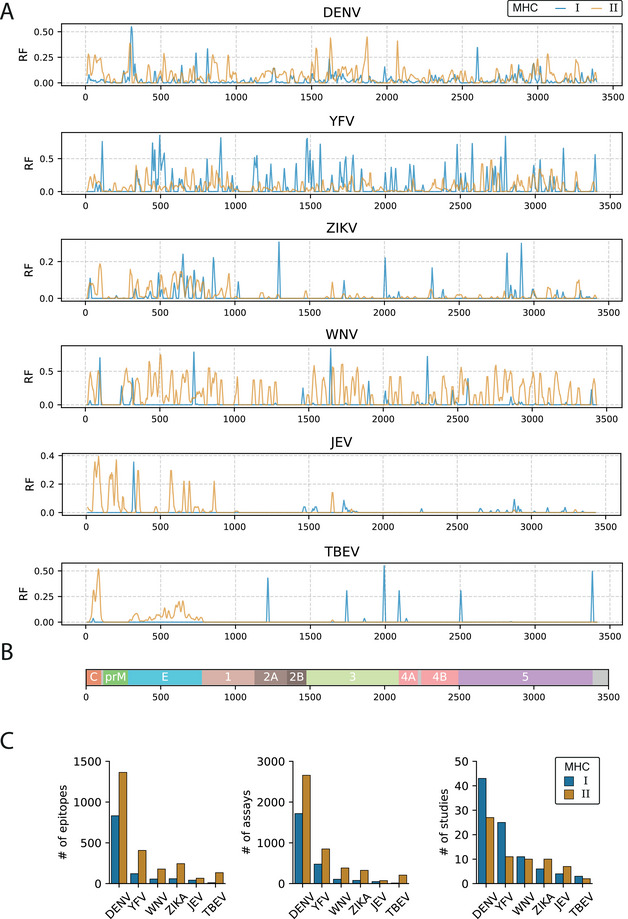
Immunodominant regions across the flavivirus proteome. (A) Data on immunodominant regions of flaviviruses were retrieved from the IEDB Immunome Browser tool (www.iedb.org). The response frequency (RF) represents the number of individuals with a positive response divided by the number of individuals tested to a peptide at a given residue. Lower‐bound values were chosen and recalculated as a moving average over 10 amino acids for visualization purposes. (B) Amino acid positions across the proteome. (C) Bar graphs showing the number of epitopes, assays, and studies for each flavivirus obtained from the IEDB Immunome Browser tool.

In this context, we retrieved data from the Immune Epitope Database and Tools [15] (IEDB, www.iedb.org) to identify and characterize experimentally validated epitopes across the entire proteome of six flaviviruses (Figure 2A). In this database, epitopes are presented as response frequency (RF), the number of subjects who responded divided by the number of subjects tested. Generally, identified epitopes are more abundant in specific regions, with immunodominant patterns emerging in capsid, envelope, NS1, NS3, and NS5 proteins (Figure 2B). Notably, considerable variation exists in the number of identified epitopes for each virus, largely reflecting the uneven availability of studies for the different flaviviruses, most of which focus on dengue (Figure 2C). This discrepancy underscores the need for further mapping of epitopes across different flaviviruses. A list of the immunodominant epitopes from IEDB included in Figure 2A, sorted by response frequency >80% in at least two responders, is provided in Table 1.

**TABLE 1:** Immunodominant epitopes of flaviviruses.

IEDB Epitope ID^a^	Sequence	Position	Protein	HLA Allele
73285	WYMWLGARY	2967‐2975	NS5	HLA‐A*01:01, HLA‐A*24:02, HLA‐A*24:03, HLA‐A*29:02, HLA‐A*30:02
22824	GTSGSPIVDR	1608‐1617	NS3	HLA‐A*11:01, HLA‐B*57:01
34918	LAPTRVVAAEME	1697‐1707	NS3	HLA‐B7
133501	AIVREAIKR	1682‐1690	NS3	HLA‐A*02:01
133716	TLYAVATTI	2284‐2292	NS4B	HLA‐A*02:01, HLA‐A*02:03, HLA‐A*32:01, HLA‐B*35:01
174066	CGGLKNVREVKGLTKGG	2584‐2600	NS5	HLA‐A2
46576	NYADRRWCF	2033‐2040	NS3	HLA‐A*23:01, HLA‐A24, HLA‐A*24:02
183619	HTWTEQYKF	801‐809	NS1	HLA‐A*32:01, HLA‐B57, HLA‐B*57:01
65552	TPEGIIPTL	1976‐1984	NS3	HLA‐A*01:01, HLA‐A*02:01, HLA‐A*03:01, HLA‐A*11:01, HLA‐A*31:01, HLA‐A*69:01, HLA‐B*07:02, HLA‐B*15:01, HLA‐B*27:05, HLA‐B35, HLA‐B*35:01, HLA‐B*40:01, HLA‐B*58:01
121572	LLWNGPMAV	2470‐2478	NS4B	HLA‐A*02:01, HLA‐A2
110040	VLAGWLFHV	1471‐1479	NS2B	HLA‐A*02:01, HLA‐A*02:05, HLA‐A*69:01, HLA‐B*15:42, HLA‐B*45:06, HLA‐B*83:01, HLA‐C*04:01, HLA‐C*06:02
232024	KSEYMTSWFY	2792‐2801	NS5	HLA‐A*01:01
180343	RPIDDRFGL	3178‐3186	NS5	HLA‐B*07:02, HLA‐B*35:03, HLA‐B7
606315	TESWIVDRQW	485‐494	E	HLA‐B*44:02, HLA‐B*44:03
232480	YTDYLTVMDRY	3389‐3399	NS5	HLA‐A*01:01
232463	YMWLGARY	2983‐2990	NS5	HLA‐A*01:01, HLA‐A*29:02, HLA‐B*15:01, HLA‐B*35:01
46075	NTDIKTLKF	438‐446	E	HLA‐A*01:01, HLA‐A*80:01
62179	SVKEDLVAY	1557‐1565	NS3	HLA‐A*25:01, HLA‐A*26:01, HLA‐B*15:01, HLA‐B*35:01
231390	RPIDDRFGLAL	3178‐3188	NS5	HLA‐B*07:02, HLA‐B*35:01, HLA‐B*35:03, HLA‐DQA1*01:02/DQB1*05:01, HLA‐DQA1*01:03/DQB1*06:03, HLA‐DQA1*02:01/DQB1*03:01, HLA‐DQA1*05:01/DQB1*03:02, HLA‐DRA*01:01/DRB1*04:04, HLA‐DRA*01:01/DRB1*09:01, HLA‐DRA*01:01/DRB1*14:54, HLA‐DRA*01:01/DRB3*01:01
232306	SPRERLVLTL	1227‐1236	NS2A	HLA‐B*07:02, HLA‐B*08:01
23582	HAVPFGLVSM	1134‐1143	NS2A	HLA‐B35, HLA‐B*35:01, HLA‐B*35:02, HLA‐B*35:03, HLA‐B*35:08
606319	TRRFLPQIL	1689‐1697	NS3	HLA‐C*06:02
24446	HPFALLLVL	1464‐1472	NS2B	HLA‐B*07:02, HLA‐B35, HLA‐B*35:01, HLA‐B*35:02, HLA‐B*35:03, HLA‐B7
231947	HESHLVRSW	1119‐1127	NS1	HLA‐B*15:01, HLA‐B*44:02, HLA‐B*44:03
33914	KTWGKNLVF	894‐902	NS1	HLA‐A*32:01, HLA‐B*15:01, HLA‐B*15:17, HLA‐B*57:01, HLA‐B*58:01
231240	MPEAMTIVML	2154‐2163	NS4A	HLA‐B*07:02, HLA‐B*35:01, HLA‐B*35:03, HLA‐B*53:01, HLA‐DRA*01:01/DRB3*03:01, HLA‐DRA*01:01/DRB5*01:01
606250	DVILPIGTR	2762‐2770	NS5	HLA‐A*33:01, HLA‐A*68:01
606298	RIRDGLQYGW	884‐893	NS1	HLA‐A*32:01, HLA‐B*57:01
14311	ESWIVDRQW	486‐494	E	HLA‐B*15:17, HLA‐B*57:01, HLA‐B*58:01
606293	QEVEFIGY	453‐460	E	HLA‐B*18:01
73285	WYMWLGARY	2982‐2990	NS5	HLA‐A*01:01, HLA‐A*24:02, HLA‐A*24:03, HLA‐A*29:02, HLA‐A*30:02
58348	SHDVLTVQF	102‐110	C	HLA‐A*01:01, HLA‐A*02:01, HLA‐A*03:01, HLA‐A*11:01, HLA‐A*29:02, HLA‐A*31:01, HLA‐A*69:01, HLA‐A*80:01, HLA‐B*07:02, HLA‐B*15:01, HLA‐B*15:09, HLA‐B*27:05, HLA‐B*38:01, HLA‐B*39:01, HLA‐B*40:01, HLA‐B*44:02, HLA‐B*46:01, HLA‐B*48:01, HLA‐B*57:01, HLA‐B*58:01, HLA‐C*04:01
16102	FHERGYVKL	2571‐2579	NS5	HLA‐A*01:01, HLA‐A*02:01, HLA‐A*03:01, HLA‐A*11:01, HLA‐A*29:02, HLA‐A*31:01, HLA‐A*69:01, HLA‐A*80:01, HLA‐B*07:02, HLA‐B*15:01, HLA‐B*15:09, HLA‐B*18:01, HLA‐B*27:05, HLA‐B*38:01, HLA‐B*39:01, HLA‐B*40:01, HLA‐B*44:02, HLA‐B*46:01, HLA‐B*48:01, HLA‐B*57:01, HLA‐B*58:01
606324	VEFEPPHAA	517‐525	E	HLA‐B*40:02, HLA‐B*50:01
606314	SVAGRVDGL	1397‐1405	NS2B	HLA‐A*02:05
606270	HEMYYVSGA	2723‐2731	NS5	HLA‐B*50:01
232289	SMMPEAMTI	2152‐2160	NS4A	HLA‐A*02:01, HLA‐A*32:01, HLA‐B*15:01, HLA‐B*52:01
606269	HDVLTVQF	103‐110	C	HLA‐B*37:01
606254	ETVAIDRPA	331‐339	E	HLA‐A*68:02
1311573	LPVWLSWQVA	2030‐2039	NS3	HLA‐B*56:01
62139	SVGGVFTSV	720‐728	E	HLA‐A*02:01
59089	SLFGQRIEV	2288‐2296	NS4B	HLA‐A*02:01
22823	GTSGSPIVDK	1638‐1647	NS3	HLA‐A11, HLA‐A*11:01
187063	GTLTSAINR	89‐97	C	HLA‐A*11:01
42299	MPNGLIAQF	2006‐2014	NS3	HLA‐B35
57986	SGATWVDLV	306‐314	E	*NA*
240808	ILLDNITTL	1984‐1992	NS3	HLA‐A*02:01, HLA‐A2
910616	MLLQAVFEL	1207‐1215	NS2A	HLA‐A*02:01, HLA‐A2
910621	NIWGAVEKV	3374‐3382	NS5	HLA‐A*02:01, HLA‐A2

^a^ Available at www.iedb.org.

### 1.3 T Cell Responses to Flavivirus Vaccines

In this section, we summarize clinical studies characterizing T cell responses to licensed and developing flavivirus vaccines. The discussion is divided based on the two major empirical vaccine approaches used for flavivirus vaccine development: live and inactivated (killed) vaccines. Live virus vaccines contain weakened (attenuated) forms of the whole virus that can infect and replicate in host cells to stimulate an immune response. These vaccines typically generate a potent and long‐lasting immune response. In contrast, inactivated vaccines encompass viruses that have been chemically or physically treated to render them incapable of replication. This distinction of different vaccine types is important, as it affects how viral antigens are presented to the immune system, ultimately shaping the type of adaptive immune response elicited. In Box 1, an overview of the central role of T cells in these vaccine‐induced responses is provided. Building on the foundational concepts discussed for flavivirus vaccines, Box 2 further explores the role of T cells in protection against viral infections by highlighting key lessons learned from the extraordinary body of research on the causative agent of the coronavirus disease 2019 (COVID‐19) pandemic, the SARS‐CoV‐2 RNA virus. This comparison underscores how insights from SARS‐CoV‐2 can deepen our understanding of T cell‐mediated immunity and inform future vaccine design.

**BOX 1 | The role of T cells in vaccine‐induced immunity**

Despite growing evidence that T cells play a crucial role in protection against viral infections, their contribution to vaccine‐induced immunity remains less studied. This is largely due to the historical and empirical nature of vaccine development, which has traditionally focused on eliciting antibody responses. The goal of vaccination is to generate long‐lived immunological memory against a specific virus. Once an individual who has been vaccinated becomes infected, the immune system rapidly recognizes viral antigens and mounts an adaptive response in a short time that controls infection and prevents disease. Key components of immunological memory are T cells, specifically virus‐specific CD4+ and CD8+ T cells, which circulate throughout the body, primed for rapid activation upon encountering viral antigens. Memory T cells are often categorized by their tissue distribution and migration patterns: effector memory T cells (Tem) circulate in the blood, central memory T cells (Tcm) reside in secondary lymphoid organs, and tissue‐resident memory T cells (Trm) remain in non‐lymphoid tissues. Immunophenotyping of memory T cells typically relies on flow‐cytometry‐based methods to detect surface markers CD45RA and CCR7, distinguishing four primary populations: naive (CD45RA+CCR7+), Tcm (CD45RA‐CCR7+), Tem (CD45RA−CCR7−), and Tem re‐expressing CD45RA (Temra, CD45RA+CCR7−) [135]. Vaccine‐induced immunological memory supports viral clearance by rapidly reducing viral load upon infection [136]. Once memory T cells are established by vaccination, they can be promptly reactivated to control viral replication after subsequent infection. Following innate immune recognition of vaccine antigens, effector memory T cells respond to virus‐derived peptides presented on MHC molecules. CD4+ T cells play a central role in orchestrating the adaptive immune response, bridging cellular and humoral immunity. Several subsets of CD4+ T cells are important for combating viral infections. Th1 cells mediate antiviral functions through the secretion of IL‐2, TNF‐α, TNF‐β, and IFN‐γ, which stimulates CD8+ T cells. Tfh cells reside in secondary lymphoid organs, where they promote virus‐specific B cell maturation within germinal centers, enhance antibody affinity maturation, and support the differentiation of memory B cells. These Tfh cells express the chemokine receptor CXCR5, and CXCR5+ memory Tfh cells are the dominant phenotype for providing B cell help and mediating recall antibody responses [137]. Although CD4+ T cells are most often recognized for their supporting role, they can also exhibit cytotoxic activity, as observed during DENV infection [138]. CD8+ T cells directly contribute to viral clearance by inducing apoptosis of infected cells and producing pro‐inflammatory cytokines. In summary, multiple subsets of T cells are needed for effective viral clearance, and their activation is closely tied to the availability of vaccine antigens and how the antigens are presented to the adaptive immune system through vaccination.

**BOX 2 | Lessons learned from T cell immunity to SARS‐CoV‐2 infection and vaccination**

Our experience with the COVID‐19 pandemic, caused by SARS‐CoV‐2, has significantly advanced our understanding of virology, immunology, and vaccinology, as the most studied virus in history [139]. Of importance, it has highlighted the critical role of T cell responses in protection against viral disease, as excellently reviewed elsewhere [70, 140, 141]. Clinical studies of COVID‐19 patients have revealed key aspects of T cell immunity, offering crucial insights that should be broadly applicable to other RNA viruses, including flaviviruses. Some of these insights are highlighted below. Firstly, CD8+ T cells play a crucial role in reducing viral load to prevent disease progression. In rhesus macaques, protection against SARS‐CoV‐2 challenge was achieved through convalescent serum transfer and depletion of CD8+ T cells prior to re‐challenge partially abrogated this protection, suggesting that CD8+ T cells are important to control infection [142]. Clinical studies on COVID‐19 patients with hematological cancers, who have impaired humoral immunity due to B‐cell‐depleting medication, have shown that the presence of CD8+ T cell responses is associated with higher survival rates [143]. In healthy individuals, an immunodominant CD8+ T cell epitope (NP_105‐113_‐B*07:02) has been shown to be strongly associated with less severe COVID‐19 [144]. Furthermore, individuals with CD8+ T cells targeting conserved coronavirus epitopes are more prone to developing mild COVID‐19 symptoms compared with those lacking these epitopes [145]. From these studies, a clear picture emerges: CD8+ T cells are protective against disease and important in both healthy and immunodeficient individuals with impaired antibody responses. Secondly, T cells target a broad range of epitopes across both structural and non‐structural proteins. However, certain viral proteins are more readily targeted than others. Both CD4+ and CD8+ T cells target structural proteins, which include antibody targets such as the major structural and surface spike protein [146, 147]. Indeed, spike‐specific CD4+ T cells correlate with the magnitude of IgG and IgA responses [148]. This broad and specific targeting, which also overlaps with antibody responses, underscores the importance of considering all components of the adaptive immune response for protection against viral disease. Thirdly, the SARS‐CoV‐2‐specific CD4+ T cell response is driven by Th1 cells and T follicular helper cells (Tfh). Th1 cells are crucial during viral infections as they mediate killing of virally infected cells by cytotoxic innate cells and CD8+ T cells through the release of anti‐viral cytokines. [149]. IFN‐γ is produced by both CD4+ and CD8+ T cells [150] and has been identified as a critical mediator in controlling SARS‐CoV‐2 infection in the upper airways [151, 152]. SARS‐CoV‐2‐specific Tfh cells correlate with antibody neutralization and may be directed against multiple structural proteins [153]. Lastly, the SARS‐CoV‐2‐specific T cell response displays considerable cross‐reactivity. Several studies have shown that individuals have pre‐existing SARS‐CoV‐2 reactive T cells [154]. This is likely driven by previous exposure to human coronaviruses such as OC43 [155], although it may also originate from non‐related viruses such as CMV [156]. There is evidence that the cross‐reactive T cells have clinical implications, being able to protect against severe COVID‐19 [157]. The cross‐reactive T cell responses observed between SARS‐CoV‐2 and human coronaviruses are likely due to high sequence homology, a feature that is also prevalent among flaviviruses. Furthermore, T cell responses induced by either infection or vaccination generate cross‐reactive responses to significantly mutated SARS‐CoV‐2 variants [158, 159, 160]. This highlights the ability of T cells to be less affected by the inherent genetic instability of RNA viruses, which frequently mutate epitopes in structural antigens to escape antibody recognition. The COVID‐19 vaccine landscape includes a diverse range of vaccine types, with varying degrees of vaccine efficacy and immunogenicity depending on vaccine platform [161]. The differences in vaccine efficacy across vaccine platforms are likely correlated with the distinct immune responses they elicit. Among the most used COVID‐19 vaccines are the mRNA BNT162b2 (Pfizer‐BioNTech) and inactivated CoronaVac (Sinovac Life Sciences). Clinical phase 3 trials have estimated their vaccine efficacy to be 95% and 83.5% [162, 163], respectively. A large clinical study showed that the risk of symptomatic infection, hospitalization, and severe disease from COVID‐19 was greater in those who received inactivated vaccines compared to mRNA vaccines [164]. Several studies have shown different capacities to induce immune responses when comparing mRNA and inactivated COVID‐19 vaccines, which could potentially explain the observed discrepancies in vaccine efficacy. A hallmark of mRNA vaccines is their ability to elicit strong, neutralizing, and long‐lasting spike‐specific antibodies, as well as robust spike‐specific memory T cell responses [70], which include effector CD4+ T cells such as Th1 cells and Tfh cells, along with CD8+ T cells [165]. Studies have shown that spike‐specific antibody response is lower after inactivated vaccination compared to mRNA vaccination [166, 167]. Most studies comparing CD4+ T cell responses have found lower responses following inactivated vaccination relative to mRNA vaccination [167, 168]. However, in an age‐matched comparison, the inactivated vaccine elicited higher CD4+ and CD8+ T cell responses to structural antigens [78]. It has been shown that Omicron‐specific CD4+ and CD8+ T cell responses in people aged 60 years or older are lower following inactivated vaccination compared with mRNA vaccination [168], which could explain the lower vaccine protection observed [169]. Despite potential differences in the immune response between inactivated and mRNA vaccines against COVID‐19, inactivated vaccines were highly effective in preventing COVID‐19 [170] and generated diverse T cell responses, including memory Tfh cells that correlate with neutralizing antibody responses [171]. The large and diverse vaccine landscape of SARS‐CoV‐2 vaccines allows for direct comparisons between platforms, offering unique insights into T cell responses and vaccine development. The lessons learned from the different immune responses driven by these vaccine platforms are numerous and should inform future flavivirus vaccine development.

## 2 Live Attenuated Flavivirus Vaccines

The immune response to live attenuated vaccines closely resembles that of a natural mild infection, engaging all major components of the immune system. This makes them highly immunogenic and capable of inducing long‐lasting immunity. Their strong immunogenicity is driven by multiple factors, beginning with potent stimulation of the innate response through the activation of a variety of pattern recognition receptors (PRRs). Because they retain infectivity, live attenuated vaccines present antigens to stimulate diverse immune responses. This includes robust T cell activation, as intracellular antigens are processed by infected cells and presented on MHC class I molecules to CD8+ T cells. Furthermore, replication leads to high and prolonged antigen persistence, which continuously stimulates the immune system, allowing for sustained priming of the adaptive immune system [16]. A large number of clinical studies have characterized T cell responses induced by licensed live attenuated YFV and DENV vaccines, whereas data for the live attenuated JEV SA14‐14‐2 vaccine [17] are limited and therefore not discussed further.

### 2.1 YFV Vaccine

The YFV vaccine was developed in the 1930s through attenuation of the patient isolate Asibi, resulting in the 17D vaccine strain, which remains in use today with multiple subtypes available. The history of the YFV vaccine provides valuable historical context and is reviewed elsewhere [18]. The YFV vaccine is considered one of the most effective vaccines ever developed. Despite being developed before the advent of modern randomized controlled clinical trials, numerous studies have demonstrated its protective ability in humans [19]. This includes seroconversion rates exceeding 90% and lasting over 30 years [20]. A recent meta‐analysis confirmed the long‐lasting immunity conferred by YFV vaccination, with a 94% seroprotection rate in healthy adults, with life‐long protection achieved after a single dose [21].

The role of T cells in the immune response to YFV vaccination has been studied for over 30 years, beginning with the observation that vaccination activates CD8+ T cells [22, 23]. Since then, evidence has demonstrated that CD8+ T cells play a crucial role in protection against YFV by reducing viral load [24], which is associated with lower mortality rates [25]. Further studies have shown that both CD4+ and CD8+ T cells contribute significantly to the immune response following YFV vaccination [24, 26, 27, 28, 29, 30, 31, 32, 33, 34, 35, 36, 37, 38, 39, 40]. Specifically, YFV vaccination results in a potent activation and proliferation of CD4+ and CD8+ T cells, which can be identified using activation markers such as CD154 (CD40L), CD38, and HLA‐DR, as well as the proliferation marker Ki‐67 [26, 28]. Activated T cells exhibit polyfunctionality with expression of multiple antiviral cytokines. Among CD4+ T cells, both T helper 1 (Th1) and T helper 2 (Th2) responses are induced, with cytokine secretion of IL‐2 and interferon (IFN)‐γ, as well as IL‐5 and IL‐13, respectively [27, 28]. CD8+ T cells express multiple markers associated with degranulation (CD107a) and several cytokines, including IFN‐γ, interleukin (IL)‐2, and tumour necrosis factor (TNF)‐α, as well as the chemokine macrophage inflammatory protein (MIP)‐1β [30, 41]. Longitudinal studies have elucidated the kinetics of the T cell response, which peaks around 14 days after vaccination [29], with CD4+ T cells preceding the CD8+ T activation [41]. Although the T cell response begins to wane after 2 weeks, long‐lasting responses have been detected years after vaccination [29, 34]. The generation of long‐lived memory T cells is a hallmark of YFV vaccination and a key factor in its life‐long protective efficacy. Studies have identified a YFV‐specific T cell population with naive‐like phenotype, referred to as stem cell‐like memory T (TSCM) cells, as a source of durable memory, which have been shown to persist for up to 25 years post‐vaccination [34]. A recent study revealed that multiple memory subsets, including TCSM cells, of YFV‐specific CD4+ T cells are generated months after vaccination, providing additional clues to the complexity of long‐lasting T cell immunity [40].

Immunogenic CD4+ and CD8+ T cell epitopes have been identified across all ten structural and non‐structural YFV proteins in different HLA contexts, demonstrating the broad T cell response elicited by YFV vaccination [23, 30, 31, 32, 38]. There is an overlap of targets, with epitopes across multiple viral proteins, including envelope, NS3, and NS5, which are recognized by both CD4+ and CD8+ T cells [32]. Certain epitopes are more frequently recognized than others, with immunodominant responses often being HLA‐restricted. Several studies have identified the HLA‐A2‐restricted CD8+ epitope LLWNGPMAV within the NS4b protein, which is detected in most HLA‐A2‐positive vaccine recipients [30, 41]. This has enabled in‐depth analysis of epitope‐specific responses using tetramer staining, revealing that the LLWNGPMAV‐specific T cell population can constitute as much as 17% of all CD8+ T cells at peak response and persist for at least one year post vaccination [42]. However, whether immunodominant epitope‐specific T cells exhibit superior protective capacity compared with non‐immunodominant T cell responses remains unclear.

Although neutralizing antibodies are considered a key correlate of protection for YFV vaccination, a recent study demonstrated that vaccine‐induced T cell responses can control viral replication in the absence of such antibody responses [43]. Notably, T cells specific to the capsid protein were identified as the primary mediators of this protective effect. Similar findings have been reported for SARS‐CoV‐2, where a study demonstrated that prior mRNA COVID‐19 vaccination protected mice against SARS‐CoV‐2 challenge independently of antibodies [44]. These findings are intriguing and should stimulate further interest in understanding the role of the vaccine‐induced T cell responses and their protective mechanisms in broader contexts.

Immunophenotyping studies of vaccinated individuals have revealed substantial variations in the immune response, with the induction of T cells being highly heterogeneous. A recent study found that HLA type and baseline frequencies of activated CD4+ and CD8+ T cells distinguish weak and good vaccine responders, among other factors [45]. Understanding the source of variation in the vaccine response is crucial for developing effective vaccines that provide broad protection, irrespective of genetic or environmental factors. However, further studies are needed in this context and would be important as a step towards personalized vaccine strategies.

### 2.2 Chimeric YFV Vaccines

The extraordinary immunogenicity and efficacy of the YFV vaccine have led to the development of recombinant live vaccines using YFV as a vector, both for other flaviviruses (e.g., for ZIKV and WNV) and for non‐flaviviral pathogens (e.g., Ebola virus and SARS‐CoV‐2) [46]. These recombinant vaccines are generated by replacing the pre‐membrane and envelope proteins of the YFV backbone with those of the target virus, resulting in a chimeric vaccine that retains the capsid and NS proteins of YFV. Currently, licensed YFV‐based recombinant vaccines include those against JEV and DENV.

### 2.3 CYD‐TDV

It is estimated that over half of the world's population is at risk of DENV infection, with approximately 100 million people developing symptomatic infections each year [47]. CYD‐TDV (Dengvaxia in EU, Sanofi Pasteur) was the first licensed vaccine against dengue and its four serotypes (DENV1‐4). However, its use has been restricted to individuals who have had a prior DENV infection and are over the age of 9 years. This limitation is due to the observed increased risk of severe disease in individuals who experience a secondary DENV infection with a different serotype, a phenomenon linked to the immunological mechanism known as antibody‐dependent enhancement (ADE) [48]. Furthermore, protection conferred by CYD‐TDV has been moderate, with phase 3 clinical trials reporting vaccine efficacy ranging from 57% to 61%, varying by serotype and serostatus [49, 50]. The moderate vaccine efficacy has been hypothesized to be related to the absence of non‐structural proteins in CYD‐TDV, which would otherwise stimulate protective DENV‐specific CD8+ T cell responses. This is a critical component absent in the T cell response, since a protective role for CD8+T cells in DENV‐infected patients has been demonstrated [51]. Additionally, the lack of capsid‐specific T cells in the vaccine may also contribute to reduced adaptive immune responses and protection [52]. This was further supported by findings from a study of CYD‐TDV vaccine recipients, where CD4+ T cells predominantly targeted DENV envelope protein, while CD8+ T cell responses were directed only against YFV NS proteins [53]. Developing a DENV vaccine is particularly challenging as protection against all four DENV serotypes is required, and sub‐optimal responses can lead to ADE [54]. The chimeric CYD‐TDV vaccine highlights the complexity of generating broad cross‐reactive responses that retain functionality and quality that protect against infection.

### 2.4 TAK‐003

A second licensed vaccine against DENV is TAK‐003 (Qdenga in the EU, Takeda Vaccines), a live attenuated tetravalent DENV vaccine based on a DENV2 backbone. In a phase 3 clinical trial, the study endpoint was met, demonstrating a favorable safety profile and an efficacy of 80% [55]. A recent 4.5‐year follow‐up study demonstrated that the vaccine provides protection against all four DENV serotypes in both previously exposed and DENV‐naive individuals [56]. A comprehensive study on T cell responses following TAK‐003 vaccination demonstrated that vaccine‐induced CD4+ and CD8+ T cells targeted all four DENV serotypes, were multifunctional, and persisted for up to three years post‐vaccination [57].

### 2.5 TV003 and TV005

The US National Institutes of Health (NIH) has been developing a live attenuated DENV vaccine for over 20 years and is currently in clinical trials as two tetravalent formulations (TV003 and TV005) [58, 59]. The first detailed study on cellular responses was investigated in recipients who either received monovalent or tetravalent DENV vaccination, and it was shown that the T cell response varied [60]. The response after monovalent vaccination was driven by IFN‐γ+ T cells directed against both structural and NS proteins, with the majority of responses against NS (63%–90%). In contrast, the T cell response after tetravalent vaccination was almost entirely directed against NS proteins (99.8%). Finally, the T cell response to tetravalent vaccination was mainly directed against conserved epitopes in the NS3 and NS5 regions, and the memory response was mainly driven by effector memory (Tem) and effector memory cells re‐expressing CD45RA (Temra) T cell subsets. Human challenge model studies are rare and can offer highly detailed characterizations of immune responses. An attenuated DENV2‐based human challenge model was used to show that TV003 is protective [61] and elicits virus‐specific T cell responses [62]. The response was characterized by multifunctional CD4+ and CD8+ T cells expressing IFN‐γ and TNF‐α, with CD4+ T cells responding earlier than CD8+ T cells. Primary immunization resulted in the expansion of Tem cells, whereas booster vaccination or challenge with DENV2 led to the expansion of Temra cells, which were maintained for at least 1 year post‐vaccination. Detailed characterization of the CD4+ T cell response to TV005 demonstrated a more equal response toward structural (40%) and NS (60%) proteins [63]. The CD4+ T response was dominant towards capsid, and overall, T cell epitopes extensively overlapped with epitopes detected after natural infection. The demonstrated protective role of T cells against dengue disease is promising and should inform the development of future flavivirus vaccines, as it is reasonable to assume that all flavivirus vaccines would benefit from inducing T cells capable of controlling viral infection. Building on these insights, new vaccine strategies are in development, including T‐cell‐based vaccines that selectively induce T‐cell responses without stimulating antibody production, thereby avoiding ADE [64]. This includes vaccine candidates against ZIKV based on immunodominant epitopes across NS proteins [65] or NS3 [66, 67], which have been demonstrated in mice to protect against ZIKV challenge in the absence of antibody responses.

## 3 Inactivated Flavivirus Vaccines

Inactivated vaccines have been used for over a century to provide effective protection against viral diseases. They are more technically straightforward to develop and produce, while at the same time offering a safe alternative to live attenuated vaccines for immunocompromised individuals. The main effector mechanism in the immune response induced by inactivated vaccines is mostly attributed to binding and neutralizing IgG antibodies. The T cell response induced by inactivated vaccines is mainly limited to CD4+ T cell responses since these vaccines do not efficiently enter the MHC class I presentation pathway, consequently resulting in low CD8+ T cell activation.

There is evidence to suggest that a limited capacity to induce T cell responses is a common feature of inactivated vaccines. Comparative studies have demonstrated that, unlike inactivated vaccines, live attenuated influenza vaccine [68] and SARS‐CoV‐2 mRNA vaccines [69] activate and generate efficient memory CD8+ T cell responses. Most studies of inactivated SARS‐CoV‐2 vaccines followed the expectation that only CD4+ T cell responses were induced [70], including large clinical studies [71, 72, 73, 74] and in‐depth studies using single‐cell RNA sequencing [75]. However, it should be noted that these observations have, in part, been contradicted by some studies that were able to measure CD8+ T cell responses [76, 77, 78], showcasing the need for more research in this area. Future studies should address the molecular mechanisms driving these diverging immune responses measured in different studies and determine whether they are driven by confounding factors related to differences in study cohort characteristics and varying methodological approaches used to measure T cells.

The limited antigen content and breath comprised in inactivated vaccines necessitate multiple doses to achieve protection, and booster doses are required to maintain protection. To enhance immunogenicity, adjuvants are often included in inactivated vaccine formulations to improve both antibody and cellular immune responses. Highly effective inactivated vaccines are available for TBEV and JEV, demonstrating their value in preventing flavivirus‐associated diseases.

### 3.1 TBEV Vaccines

Tick‐borne encephalitis virus (TBEV) is a major pathogenic flavivirus capable of causing neurological disease in humans. It is transmitted to humans through the bite of an infected tick, primarily from *Ixodid* species, which are distributed across endemic countries [79]. The most effective strategy to control and prevent TBE is vaccination [80]. All current licensed TBEV vaccines are inactivated whole‐virus vaccines based on different viral serotypes. The two European vaccines are derived from the Austrian Neudoerfl isolate, FSME‐IMMUN (Pfizer Europe), and the German K23 isolate Encepur (Bavarian Nordic). These vaccines are formaldehyde‐inactivated and aluminum hydroxide‐adjuvanted. They are considered safe, well‐tolerated, and provide effective protection. Clinical studies have demonstrated that following primary vaccination with a three‐dose regimen, 100% of vaccinated individuals develop TBEV‐specific neutralizing antibodies, which are maintained by booster immunizations [81]. A systematic literature review of studies conducted in Europe estimated over 92% vaccine efficacy of TBEV vaccines in all age groups and was protective against both mild infections and infections requiring hospitalization [82].

Few studies have examined the effects of viral antigen inactivation on T cell responses. Consequently, most studies on TBEV vaccination have focused on T cell responses targeting structural antigens. Several studies have compared T cell responses after vaccination with natural infection [83, 84, 85]. These studies have shown that both CD4+ and CD8+ T cells play important roles in controlling infection and limiting disease severity [86]. Both infected and vaccinated individuals generate CD4+ T cell responses against structural proteins, which primarily target the envelope protein, followed by the capsid protein. However, when comparing the magnitude of CD4+ T cell response, the envelope‐ and capsid‐specific CD4+ T cells were lower in infected individuals compared to vaccinated individuals [83]. Both groups generated Th1‐driven immune responses, characterized by polyfunctional cells expressing IL‐2, TNF‐α, and IFN‐γ, but with varying degrees of activation. Notably, the IFN‐γ‐producing CD4+ T cell responses were significantly lower following vaccination than after natural infection [84, 85]. A CD8+ T cell response against structural proteins was not identified in earlier studies [85]. However, more recent analysis of TCR clonotypes has demonstrated expansion of both a CD4+ and CD8+ T cell population, indicating that TBEV vaccination does induce CD8+ T cell responses [87].

A recent study provided new insights into T cell responses following TBEV vaccination by examining a cohort of vaccinated individuals and patients infected with TBEV, both with and without prior vaccination, which allowed studies of vaccine breakthrough infections [88]. The magnitude of IFN‐γ‐producing T cells assessed by ELISpot was similar for envelope‐ and capsid‐specific responses across all three groups. However, vaccinated individuals did not develop T cell responses against NS1, NS3, and NS5. The magnitude of IFN‐γ‐producing T cell responses was higher in vaccinated individuals compared to unvaccinated TBE patients. Intracellular cytokine staining further confirmed that the T cell response was mediated by both CD4+ and CD8+ T cells, with results comparable to the ELISpot data. Interestingly, TNF‐α‐producing CD4+ and CD8+ T cell responses against the capsid protein were higher in vaccinated individuals than in those who were naturally infected. In conclusion, this study demonstrated a role for functional CD4+ and CD8+ T cells following TBEV vaccination, with responses primarily directed against structural proteins [88]. Understanding the specific targets of T cell responses is critical, as illustrated by a recent study showing that poor T cell responses to envelope, NS3, and NS5 correlate with increased TBE severity [89].

### 3.2 JEV Vaccines

Japanese encephalitis is the leading cause of viral encephalitis in many countries in Asia. Vaccine development against JEV has a long history, also dating back to the 1930s, like the YFV vaccine [90]. The most widely used JEV vaccines include live attenuated JEV vaccines, inactivated Vero cell‐derived JEV vaccines, and live chimeric JEV vaccines. Due to the favorable safety profile, inactivated mouse brain‐derived vaccines were replaced by second‐generation Vero‐cell‐derived JEV vaccines, which are now widely used. Ixiaro (IC51 development name, Valneva) is an inactivated Vero‐cell‐derived JEV vaccine which is aluminum‐adjuvanted and, like the live attenuated JEV vaccine based on the SA‐14‐14‐2 virus strain [91]. Phase 3 clinical studies have demonstrated 95–100% seroconversion rates following the two‐dose primary vaccine schedule, with high seroprotective rates maintained with booster immunizations [92, 93]. Despite the widespread use of the JEV vaccine, comprehensive immunogenicity studies remain limited. The primary correlate of protection has been identified as the presence of neutralizing antibodies [94]. However, polyfunctional CD4+ and CD8+ T cell responses have been observed in JEV‐exposed individuals [95]. Additionally, IFN‐γ‐producing T cell responses have been detected following SA‐14‐14‐2 live attenuated vaccination, suggesting an important role for cellular immunity in protection against JEV [17].

Few studies have investigated the vaccine‐induced T cell response. However, in a recent study, we demonstrated that JEV vaccination elicits JEV‐specific (CD69+CD40L+) CD4+ T cells targeting the envelope and capsid proteins, as well as JEV‐specific (CD69+41BB+) CD8+ T cells directed against the envelope protein, with no detectable responses to NS5 [96].

## 4 Impact of Pre‐Existing Immunity on Vaccine‐Induced T Cell Responses

Because the number of potential pathogen‐derived peptides vastly exceeds the possible number of available T cell receptors in the human body, T cells are inherently cross‐reactive, with each T cell capable of recognizing thousands of peptides [97, 98]. Internalized and processed viral proteins, which are often associated with conserved epitopes as demonstrated by studies of influenza virus [99, 100], are presented on MHC class I molecules to CD8+ T cells, which may be the primary T cell subset involved in cross‐reactive responses [101]. It has been demonstrated that the frequency of cross‐reactive T cells, in the absence of neutralizing antibody responses, was associated with less severe disease from influenza infections during the 2009 H1N1 pandemic in the UK [102]. A major source of cross‐reactive T cells is pre‐existing immunity acquired from prior exposure to infection or vaccination. In many cases, individual T cell clones can be involved in responses to multiple infections through recognition of shared or structurally similar epitopes, leading to heterologous immunity [103]. High sequence homology, found particularly in conserved genomic regions of related viruses, may play a key role in driving cross‐reactive T cell responses. The extent of cross‐protection will likely vary among flaviviruses, both within and between different serocomplexes [104].

The topic of flavivirus cross‐reactivity gained increased interest following the 2015 ZIKV outbreak in Brazil that spread across the Americas, which spurred vaccine development for ZIKV and investigations into the immunological relationship between ZIKV and DENV due to their high sequence homology and geographical overlap. Extensive reviews on this topic are available [105, 106, 107, 108, 109]. However, the extent to which immune memory from prior flavivirus exposure shapes immune responses and clinical outcomes during secondary heterogenous flavivirus infections remains incompletely understood. Cross‐reactive immunity may be either beneficial or pathological, with ADE being a key example of potentially harmful immune interactions [110]. Below, we discuss the current understanding of cross‐reactive responses in the context of flavivirus vaccine‐induced immune responses. We make the distinction between sequential and simultaneous vaccination, which refer to the generation of heterologous immunity from a previous vaccination within an extended time frame or from a concomitant administration (co‐administration) of vaccines, respectively.

### 4.1 Cross‐Reactive Immune Responses from Previous and Sequential Flavivirus Vaccination

Several clinical trials have evaluated how pre‐existing immunity from flavivirus vaccination influences the antibody response to subsequent heterologous flavivirus vaccination [111, 112, 113, 114, 115, 116]. The findings have been somewhat inconsistent, with some studies reporting no significant impact [114], while others have demonstrated both beneficial [111, 115] and detrimental effects [112, 113, 116]. Multiple mechanisms have been proposed to explain these discrepancies, including differences in vaccine platforms and the degree of antigenic similarity between different flaviviruses [117]. However, despite variations in immunogenicity, most studies report no significant safety concerns associated with heterologous flavivirus vaccination. Nonetheless, a recent study suggested a potential risk of ADE to YFV infection following prior TBEV vaccination, indicating that prior exposure can influence infection outcomes [118].

Several studies have investigated how pre‐existing immunity from flavivirus vaccinations influences T cell responses. For instance, prior immunization with live‐attenuated JEV and DENV can induce cross‐reactive T cells that recognize ZIKV peptides [119, 120, 121]. YFV vaccination has been shown to induce CD8+ T cell responses against ZIKV NS5 [122], and there is evidence that prior JEV vaccination may also influence the YFV response [123]. However, confirming these studies will require larger study cohorts. Furthermore, both live attenuated TAK‐003 and YFV vaccines elicit cross‐reactive CD4+ and CD8+ T cell responses, as demonstrated by ex vivo stimulation of bioinformatically predicted peptides from multiple flaviviruses [124]. However, the observed T cell responses were predominantly virus‐specific, and the magnitude and functionality of cross‐reactive T cell responses were limited. Fewer studies have assessed cross‐reactive T cell responses after inactivated vaccination. In one study, individuals with prior JEV vaccination who received a purified‐inactivated ZIKV vaccine candidate exhibited a more durable CD4+ T cell response compared to those previously vaccinated with YFV [125].

There is evidence to support that flavivirus cross‐reactive T cells play a clinically significant role. For example, Grifoni et al. [121] demonstrated that previous DENV exposure led to more robust responses during subsequent ZIKV infection. Further support for the clinical importance of cross‐reactive immunity comes from studies showing that previous DENV infection confers cross‐protection against ZIKV [126, 127, 128, 129].

### 4.2 Cross‐Reactive Immune Responses from Simultaneous and Co‐administration of Flavivirus Vaccines

The findings discussed above pertain to sequential immunizations with flavivirus vaccines, where pre‐existing immunity develops over various time intervals between vaccinations. This situation differs from simultaneous vaccination, which occurs in co‐administration strategies, such as those commonly used in childhood immunization programs. The interval between exposures may significantly influence immune responses. In one study, rhesus macaques infected with ZIKV ten months prior to DENV challenge displayed higher antibody and T cell responses than those with only a 2‐month convalescence period [130]. Our recent study [131], together with work by others [132, 133, 134], demonstrated that co‐administration of different flavivirus vaccines is safe and does not adversely affect antibody responses.

Data on T cell responses from clinical studies in this context remain limited. In a recent study, we found that co‐administration of the YFV vaccine with inactivated TBEV or JEV vaccines does not significantly alter the antigen‐specific T cell response against the envelope, capsid, or NS5 proteins [96]. Furthermore, neither single nor co‐administration of these flavivirus vaccines led to notable T cell responses against ZIKV peptides from the same proteins.

**FIGURE 3:**
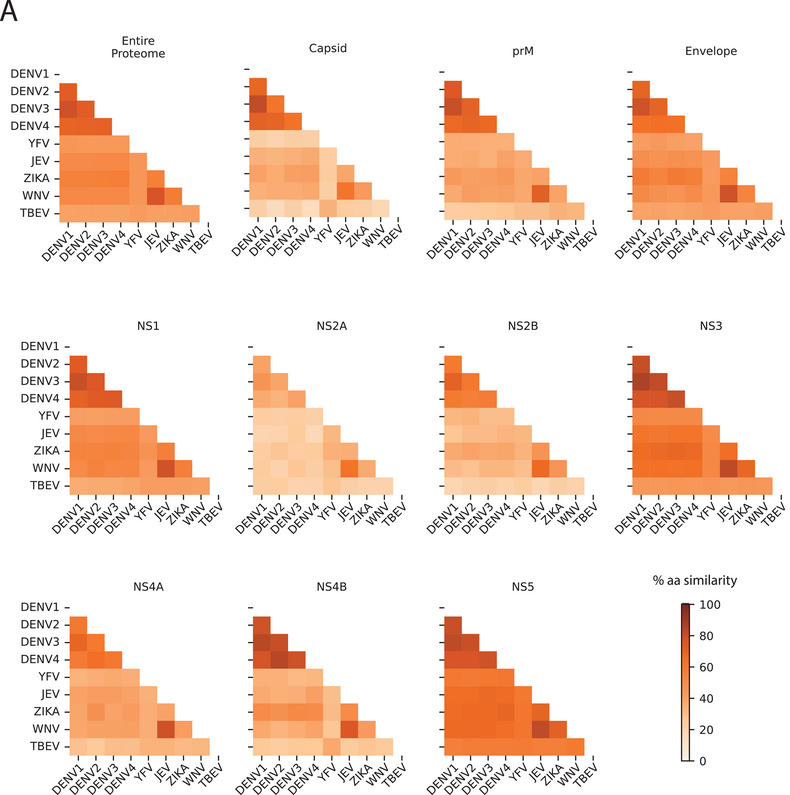
Genomic organization and pairwise distances. (A) Pairwise distance matrices, calculated as a percentage of amino acid sequence similarity, for the entire proteome and each individual protein of six flaviviruses. MEGA12 software was used to perform a ClustalW alignment, and pairwise distances were calculated on amino acids using the p‐distance method. Abbreviations: aa: amino acid, NS: non‐structural, prM: pre‐Membrane.

Taken together, findings from both sequential and simultaneous vaccination studies generate variable cross‐reactive responses between different flavivirus combinations, emphasizing the critical role of sequence similarity in driving these responses. It has been shown that an approximately 67% sequence similarity is linked to the majority (80%) of cross‐reactive T cell responses across several flaviviruses [124]. The limited cross‐reactivity between YFV and other flaviviruses is notable, as it restricts the potential to leverage the highly effective YFV vaccine to induce broad flavivirus cross‐reactive responses. This observation may be explained by phylogenetic analyses demonstrating that YFV is the most distantly related member of the flavivirus family, with fewer conserved genomic regions [104]. In Figure 3A, pairwise distances are shown based on amino acid sequence similarities for the entire proteome and individual proteins across multiple flaviviruses.

## 5 Conclusion and Perspectives

Flaviviruses remain a persistent threat to global public health, and the recent experience with the COVID‐19 pandemic highlights the devastating impact of emerging infectious diseases caused by RNA viruses. Fortunately, highly effective vaccines are available to protect against multiple flavivirus‐associated diseases, with the YFV vaccine still standing as one of the most successful vaccines developed over time. Historically, vaccine research has focused primarily on antibody responses, often overshadowing the role of T cells in vaccine‐mediated immunity. However, increasing evidence from clinical studies has demonstrated the critical role of T cells in controlling flavivirus infections and providing disease protection, as discussed in this review. This has become particularly evident in studies showing that viral infections can be controlled even in the absence of neutralizing antibodies, a phenomenon observed for both flaviviruses and coronaviruses (SARS‐CoV‐2). These findings are especially relevant for flavivirus vaccines, as T‐cell‐based vaccines could offer robust protection while mitigating the risk of ADE. The lessons learned from SARS‐CoV‐2 are numerous, but one of the most significant is the essential role that T cells play in anti‐viral immunity. Viral evolution has led to immune‐evasive mutations that reduce antibody recognition, diminishing the effectiveness of SARS‐CoV‐2 vaccines. This underscores the importance of developing flavivirus vaccines that induce strong T cell responses, which can broadly recognize viral antigens and provide durable immunity. Future flavivirus vaccines should aim to incorporate multiple viral proteins to avoid limitations in immune targeting. CD4+ and CD8+ T cells recognize both structural and non‐structural proteins, but certain T cell responses appear to be particularly important, such as YFV capsid‐specific T cells [43]. The success of mRNA‐based SARS‐CoV‐2 vaccines has demonstrated the potential to move beyond conventional live attenuated and inactivated vaccines. These next‐generation vaccines offer new opportunities for flavivirus vaccine development by allowing precise antigen selection to optimize T‐cell responses. Nucleic acid‐based vaccines, including mRNA and protein subunit vaccines, provide a more flexible and targeted approach to immune activation. Encouragingly, clinical trials evaluating mRNA vaccines against flaviviruses are already underway. The risk of emerging flaviviruses rapidly escalating into global health crises, such as recent YFV and ZIKV outbreaks, remains a significant concern. Moving forward, it will be critical to build on nearly a century of experience with flavivirus vaccines to enhance global protection. This can be achieved through improved strategies for existing licensed vaccines and the development of new vaccines, which remain our most effective defense against viral infectious diseases.

## Conflicts of Interest

The authors declare no conflicts of interest.

## Peer Review

The peer review history for this article is available at https://publons.com/publon/10.1002/eji.70027.

## Data Availability

This review article does not report any original data that has not been previously published or presented in public databases. All papers authored by the contributors and cited in this manuscript are published and available online.
